# CoDNaS 2.0: a comprehensive database of protein conformational diversity in the native state

**DOI:** 10.1093/database/baw038

**Published:** 2016-03-28

**Authors:** Alexander Miguel Monzon, Cristian Oscar Rohr, María Silvina Fornasari, Gustavo Parisi

**Affiliations:** ^1^Departamento de Ciencia y Tecnología, Universidad Nacional de Quilmes, Bernal, Argentina; ^2^Instituto de Ecología Genética y Evolución de Buenos Aires (IEGEBA)—Laboratorio de Genómica Médica y Evolución, Universidad Nacional de Buenos Aires, Argentina

## Abstract

CoDNaS (conformational diversity of the native state) is a protein conformational diversity database. Conformational diversity describes structural differences between conformers that define the native state of proteins. It is a key concept to understand protein function and biological processes related to protein functions. CoDNaS offers a well curated database that is experimentally driven, thoroughly linked, and annotated. CoDNaS facilitates the extraction of key information on small structural differences based on protein movements. CoDNaS enables users to easily relate the degree of conformational diversity with physical, chemical and biological properties derived from experiments on protein structure and biological characteristics. The new version of CoDNaS includes ∼70% of all available protein structures, and new tools have been added that run sequence searches, display structural flexibility profiles and allow users to browse the database for different structural classes. These tools facilitate the exploration of protein conformational diversity and its role in protein function.

**Database URL:** http://ufq.unq.edu.ar/codnas

## Introduction

Conformational diversity is a key concept for understanding protein function (1). Conformers are alternative structures that coexist in a dynamic equilibrium that constitutes the native state of proteins. Structural differences between conformers can be as large as relative movements of subunits or complete domains, or the rearrangements of loops and secondary structural elements (2–4). Structural differences between conformers can be as small as rotations of residues to open or close tunnels or change the shape or size of cavities and pockets (5, 6). Conformational diversity of proteins can affect biological functions such as enzyme catalysis (7), signal transduction (8), protein recognition specificity (9), promiscuity (10), allosterism (11, 12), cooperativism (13), origin of new functions and protein evolution modulation (14–16) and origin of human diseases (17). There has been a steady increase in recent years of using conformational diversity to develop new computational tools, including molecular docking (18), protein–protein interaction prediction (19), evaluation of protein structural models (20), estimation of observed substitution patterns in sequence divergence during evolution (21) and co-evolutionary measurements between residues (16).

We created a conformational diversity database of native state proteins called CoDNaS to study different aspects related to conformational diversity (22). CoDNaS is a redundant collection of 3D structures for each protein. These 3D structures include proteins that were crystallized under different conditions; for example, a protein crystallized with or without substrate, with or without post-translational modifications, or at different pH values. Since the early crystallization of hemoglobin in its oxy and deoxy forms (23), these protein structures (with the same sequence but under different conditions) could be considered as the conformational ensemble of the protein (24, 25). For each protein in CoDNaS, we performed an extensive structural comparison among all conformers and derived different structural similarity scores [root mean square deviation (RMSD), template modeling (TM) score, global distance test total score (GDT-TS) and GDT high accuracy (GDT-HA) score]. We also annotated each protein with respect to differences in the crystallization conditions for each conformer (ligands, temperature, presence of disorder, solvent accessibility and pH). CoDNaS simplifies the study of structural differences between conformers and relates these differences to biological properties that characterize the conformers (i.e. substrate unbound-bound conformers and post-translational modifications) or the protein itself (i.e. taxonomy, function and cellular location).

CoDNaS version 2 integrates extensive new upgrades into the original program. A total of 17 714 different protein chains have been included (representing a 30% increase of the original version). We also incorporated new tools for sequence searches, flexibility analyses and structural profiles, which enhance the capacity and use of the database to explore conformational diversity in proteins.

## Database construction

### Structural data sources

Different conformers for each protein were identified and extracted for the Protein Data Bank (PDB) (26) using the following protocol: BLASTClust (27) was run against all protein chains deposited in PDB to obtain all available clusters at 95% of local sequence identity with a minimum coverage of 0.90 between all sequences in the cluster. We set the limit at 95% to include putative sequence variations for a given protein. However, to avoid the inclusion of homologous sequences in a given CoDNaS entry, we used UNIPROT ID to check each cluster. The only clusters considered were those with at least two structures and with an X-ray resolution of <4.00 Å. These redundant structures of the same protein are putative conformers obtained under different experimental conditions. To estimate the structural dissimilarity between conformers, we calculated the C-alpha RMSD using MAMMOTH (28) for all possible pairs of conformers for each protein. The maximum C-alpha RMSD value for each protein entry was registered as a measure of the conformational diversity extension. All conformers for a given protein were clustered using a hierarchical procedure according to the C-alpha RMSD. Other structural measures were calculated, including TM score (29), GDT-TS and GDT-HA (30). The C-alpha RMSD per position was calculated using ProFit (31) (Martin, A.C.R. and Porter, C.T., http://www.bioinf.org.uk/software/profit/). This RMSD per position was used to derive the corresponding *Z*-score for each residue on a pair of aligned conformers. We also calculated the *Z*-scores of B-factors as a measure of intrinsic protein flexibility for each position (32).

### Biological data sources

Each conformer in CoDNaS is characterized by the experimental conditions of its structure estimation, including pH, temperature, presence of ligands, mutations, oligomeric state, post-translational modifications and presence of disorder. This information was retrieved using different data sources and our own scripts coded in PERL. The temperature, pH and sequence variation occurrence were extracted from the experimental data deposited in the PDB file. We assigned ligands using the heteroatoms in the PDB file and the BioLip database (33). The oligomeric states were provided from authors’ annotations in the PDB file and PISA (34). We used the MODRES record to extract post-translational modifications; disorder was assigned using the MobiDB database (35). All proteins in CoDNaS were linked with other biological databases using SIFTS (36). The other databases include UniProt (37); Class, Architecture, Topology and Homology (CATH) (38); Enzyme Commission (39) and Gene Ontology (40). Therefore, a user of CoDNaS can relate structural properties with biological and physicochemical information such as organism, protein function and structural classification.

### Database and web server implementation

CoDNaS was implemented on a LAMP (Linux, Apache, MySQL and PHP) server architecture. We stored most of the data in a relational database containing five tables, which was constructed using the MySQL 5.5.43 database server (www.mysql.com). Some data were retrieved from online resources like UniProt RESTful web services. The web interface was developed in HTML, CSS and JavaScript using the JQuery and Bootstrap frameworks. Server-side scripting was performed by means of PHP 5.3.3 and AJAX.

### Features and enhancements of version 2.0

CoDNaS version 2.0 has several new features and tools. The following sub-sections describe the new features and enhancements of the CoDNaS database and web-interface. For the primary usage scenario, the user can analyse the conformational diversity of a specific protein as a function of different properties such as biological function, conformer experimental conditions and taxonomy.

### Updated data

CoDNaS version 2.0 includes all protein chains derived from X-ray analysis and nuclear magnetic resonance (NMR) models as different protein conformations. The new version incorporates 263 014 new conformers belonging to 17 714 different proteins chains. These additional data effectively double the number of previously released entries. The total number of proteins covers ∼70% of PDB protein structures; the remaining 30% represents structures with resolution lower than 4.00 Å and protein clusters without two different PDB structures. There are ∼11 million total pair-wise structural comparisons between conformers for all proteins in the new database. The database can be updated semi-automatically to increase the number of protein chains and conformations with PDB updates.

### Web interface

The website was redesigned to generate a dynamic and user-friendly interface. Information is organized and shown in different sections. The user can search for proteins with different combinations of filters, and the retrieved results are shown in intuitive and informative reports. We utilized jQuery and Bootstrap frameworks to style the new design.

### Search for a protein

To facilitate database use, the CoDNaS webserver offers different search methods. The quickest method is by means of a PDB code, UniProt ID or protein name to search for a specific entry in the database. A second method (included in the Search section) allows the user to select from the following protein characteristics:

1. Extension of conformational diversity of the protein. Sets the lower and upper limits of maximum RMSD between all conformers for a given protein.
2. Causes of conformational diversity. Selects different causes of conformational diversity to retrieve proteins that present pairs of conformers associated with one or more causes.
3. Using structural information. The current version supports searching for proteins that belong to the same structural superfamily using the CATH hierarchy (41).
4. Using sequence information. The tab function 'by protein sequence' is a new feature that allows users to search by protein/DNA sequence. The server runs a BLAST query against all protein sequences in CoDNaS and retrieves proteins with a BLAST E-value better than 1E-04.

These results can be further filtered by selecting a specific experimental method (e.g. X-ray or NMR).

### Browser

CoDNaS version 2.0 supports browsing using the CATH hierarchy. The four major CATH levels can be explored by clicking on any of the class names to retrieve proteins with the same structural classification.

### Results page

**Figure 1.**
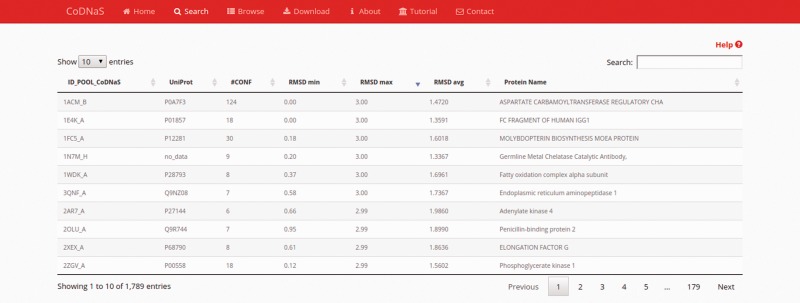
Search results page. In this example, we retrieved proteins with a maximum RMSD values between 2 Å and 3 Å, and which all conformers were obtained by X-ray diffraction. The table show the first 10 results (out 1789) and the default sorting is by ‘ID_POOL_CoDNaS’, but can be changed by clicking on any column.

After performing a protein search, the results page provides a table with the entries of retrieved proteins (Figure 1). The table contains the following seven fixed columns: protein entry ID; UniProt ID; number of conformers; minimum, average and maximum RMSD values; and protein name. Depending on the chosen search method, an extra column with CATH hierarchy can appear if the ‘browse’ section was selected, or a column with the BLAST E-value if the ‘by protein sequence’ section was selected. This table can sort the results on the basis of data in any column or text field to search a specific entry. These features enable the user to rapidly and easily select a single protein from the database. Users also can click on any row in the table to follow a link to the corresponding ‘protein entry page’ (see below).

### Protein entry page

**Figure 2.**
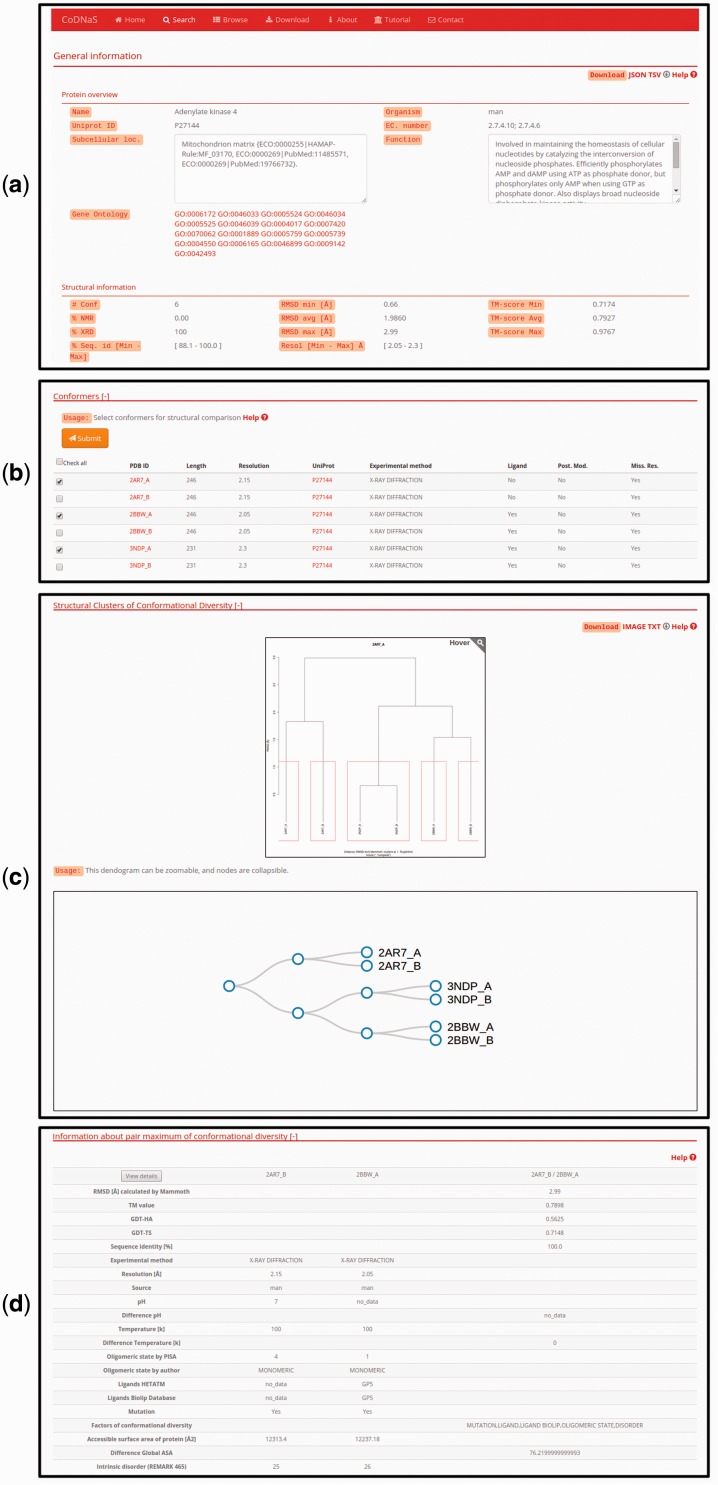
Protein entry page for Adenylate kinase 4. (**a**) Overview about biological annotations and structural information like number of conformers, experimental method, RMSD and others. All of these information can be downloaded by clicking on the download links on the top right side of the page (**b**) The detailed information for each conformer, the user can select conformers in order to retrieve all pair-wise comparison. (**c**) Hierarchical clustering using the C-alpha RMSD values between all pairs of conformers. The dendrograms shows the clusters with similar conformers, it is possible to zoom and collapse the nodes in the dendrogram on the bottom of this section. The clusters information and the image can be download with the links on the top right side on this section (**d**) Description of the maximum pair of conformational diversity of the current entry. Detailed structural information about the pair can be accessed by clicking on the button ‘View details’.

For each specific search, CoDNaS retrieves protein information as shown for the adenylate kinase 4 example in Figure 2. Adenylate kinase 4 catalyses the interconversion of nucleoside phosphates and is involved in the homeostasis of cellular nucleotides (42–44)**.** The results page includes a number of boxes that can be collapsed to optimize available workspace. The ‘General information’ box (Figure 2a) contains two sub-boxes; ‘Protein overview’ provides a basic description of the protein chain (UniProt ID, name, organism and function) and ‘Structural information’ contains a brief description of the structural alignment data (conformer resolution, experimental method, number of conformers for the protein and global sequence identity between all conformer pairs). Figure 2b shows the detailed information for each conformer, including PDB code, length and experimental method. The user can click on the checkbox to retrieve all information related to structural comparisons between selected conformations (additional details are described in the section ‘Pair-wise conformer comparison’). The next box is ‘Structural clusters of conformational diversity’, which shows the hierarchical clustering based on C-alpha RMSD values between all conformer pairs. There are two links in the top right corner of this box to retrieve the clustering dendrogram image and the corresponding text file. Finally, relevant information about the pair with maximum conformational diversity is located at the bottom of the page (Figure 2c). This box contains a table with structural information, including biological annotations and experimental conditions for the pair of conformers with maximum C-alpha RMSD in the protein. To see more details and the structural alignment of this pair of conformers, the user can click the ‘View details’ button in the top left corner of this box (additional details are described in the section ‘Pair-wise conformer comparison‘).

### Pair-wise conformer comparison

**Figure 3.**
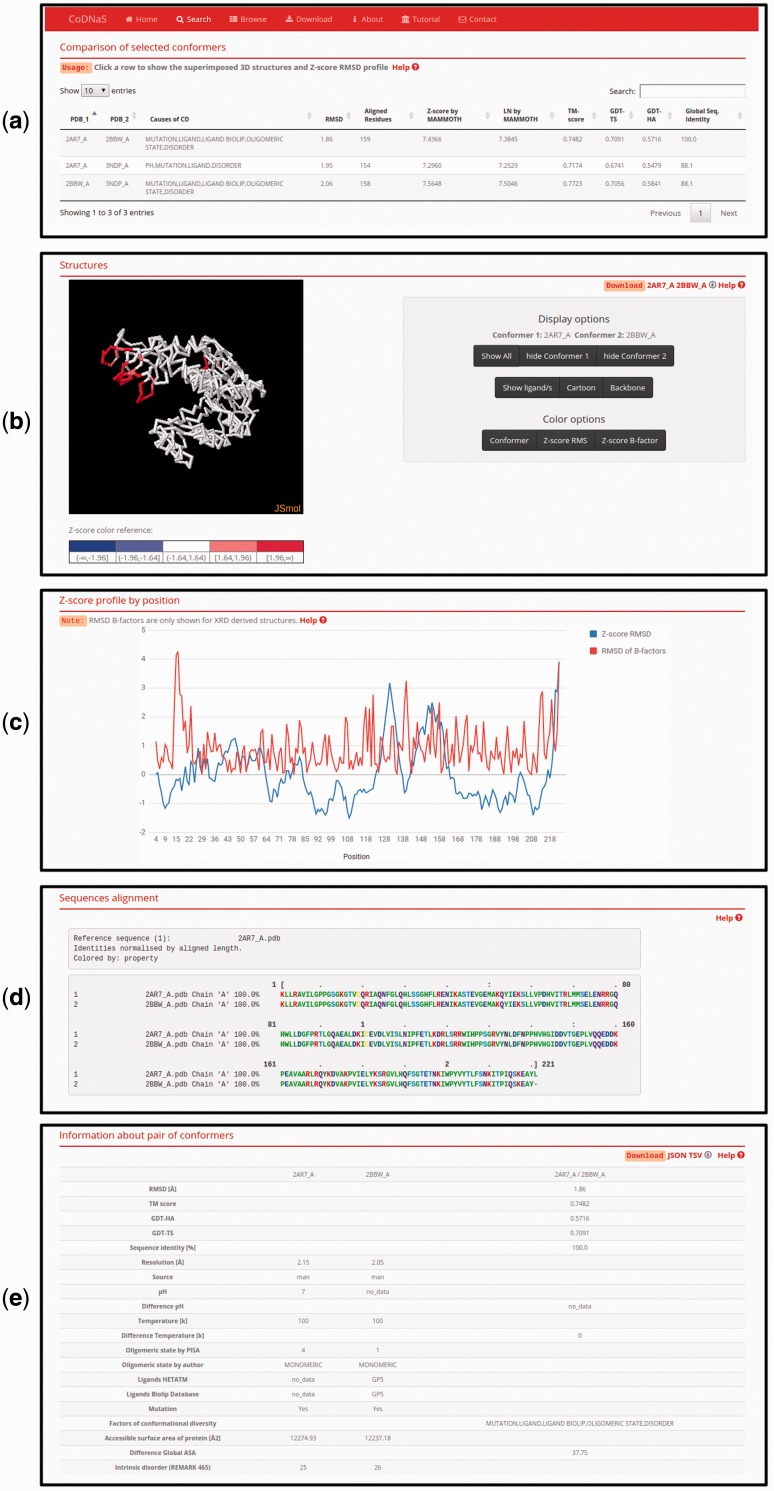
Page of pair-wise conformer comparison. (**a**) All comparisons of the selected conformers are shown in the table with the corresponding structural measures. The user can click on any pair in order to see all the information on this page. (**b**) 3D structures of each pair of conformers can be visualized. It is possible to change the colouring method of the structures (conformers, Z-score of per position C-alpha RMS and Z-score of C-alpha B-factors) like Z-score of C-alpha RMS as can be seen. (**c**) Z-score of per position C-alpha RMS and RMSD of C-alpha B-factors are plotted in the graph. (**d**) Detailed description of the current selected pair of conformers.

This new CoDNaS version 2.0 page allows the user to explore, visualize and analyse all conformer pairs for a given protein. The page contains five sections (Figure 3). The first section, ‘Comparison of selected conformers’ (Figure 3a), includes a table with all possible pairs of selected conformers with different structural data such as C-alpha RMSD, aligned residues and GDT-TS. When the user clicks on a row of this table, the web server calculates the C-alpha RMSD per residue, maps the values in the superposed PDB structures and shows four new sections. The section ‘Superimposed structures’ (Figure 3b) includes the JSmol visualization tool (45), which allows the user to display the structures of aligned conformers in 3D. The right panel contains different visualization options that allow, for example, pseudocoloring the structures according to the *Z*-score of C-alpha RMSD or the *Z*-score of B-factor values by residue. It is also possible to show ligands and change the representation of the structures.

The superimposed structures can be downloaded using the links in the top right corner of this section. Figure 3c shows an interactive plot with *Z*-score profiles derived from C-alpha RMSD per position and RMSD of C-alpha B-factors as a measure of difference in conformer flexibility (32). The user can place the pointer over a specific position in the plot to see the value in the graph. The next section, ‘Sequences alignment’, (Figure 3d) shows the sequence alignment resulting from the superposition of the selected pair of conformers. Finally, the last section, ‘Information about pair of conformers’ (Figure 3e), is a table with structural and biological information for the selected pair of conformers, including TM score, GDT, resolution, experimental conditions, presence of ligands and intrinsic disorder. This table allows users to easily explore differences between conformers and the biological implications of conformational diversity. Two download links are available in the top right corner of this section for retrieving table information.

### Download CoDNaS data

Users can search CoDNaS starting with a PDB code and retrieve all conformer pairs included in the database. The download builder allows the user to construct a downloadable file containing information of interest; data are retrieved in a tab-separated file (TSV). We also included a link to download a TSV file with all conformer pairs present in the database. Most of the sections in different views of the database allow the download of search results in TSV format or JSON (JavaScript Object Notation).

## Conclusions and future development

Understanding protein function necessarily involves understanding protein motions. CoDNaS provides a well curated, experimentally driven and thoroughly linked database of protein conformations. The capture of different conformations of the same protein allows key information to be extracted depending on protein movements, and supports the exploration of relationships between protein dynamics, experimental conditions and protein biological properties. CoDNaS includes ∼70% of all available protein structures presently deposited in PDB, and utilizes PDB updates to maintain current information for our database. Backbone and surface structural differences are very well characterized in CoDNaS, and we are planning to incorporate parameters characterizing conformational diversity largely independent of the backbone movements, such as those related to the opening and closing of tunnels and those associated with cavities or pockets. This future update will incorporate important information for these movements, which are significant for protein biological function.

## Funding

This work was supported by the Universidad Nacional de Quilmes (UNQ). G.P. and M.S.F. are researchers of the Argentinean National Research Council (CONICET). A.M.M. and C.O.R. are fellows of the Argentinean National Research Council (CONICET). Funding for open access charge: Universidad Nacional de Quilmes, grant entitled “Simulación de procesos moleculares de relevancia fisicoquímica y biológica.” and PIP-CONICET (112 201101-01002). 

*Conflict of interest*. None declared.
